# Efficacy of sertraline for post-stroke depression: A systematic review protocol of randomized controlled trial

**DOI:** 10.1097/MD.0000000000015299

**Published:** 2019-04-19

**Authors:** Zheng-fa Bai, Liu-yi Wang

**Affiliations:** a Department of Orthopedics, The Fourth People's Hospital of Shaanxi, Xi’an; b Second Ward of Neurology Department, Cardiovascular and Cerebrovascular Specialist Section of Yan’an University Affiliated Hospital, Yan’an, China.

**Keywords:** efficacy, post-stroke depression, randomized controlled trial, safety, sertraline, systematic review

## Abstract

**Background:**

Depression is a prevalent disorder for patients with stroke. Clinical researches indicate that sertraline is utilized to treat post-stroke depression (PSD) effectively. However, no systematic review has investigated this issue yet presently. Thus, this study aims to systematically assess the efficacy and safety of sertraline for patients with PSD.

**Methods:**

Literature sources will be divided into 2 sections: electronic sources and manual sources. We will search electronic literature sources as follows: EMBASE, MEDICINE, Web of Science, Cochrane Library, Allied and Complementary Medicine Database, Chinese Biomedical Literature Database, and China National Knowledge Infrastructure from their inceptions to the February 28, 2019. Manual sources include dissertations, ongoing trials, and conference abstracts. Two reviewers will select the literatures, extract and collect data information, and evaluate the risk of bias independently. Statistical analysis will be carried out by using RevMan 5.3 software.

**Results:**

Primary outcome is depression. It can be measured by Hamilton depression scale, Beck Depression Inventory, or any other scales. Secondary outcome are anxiety (as assessed by Hamilton anxiety scale, or other tools) response rate, activities of daily living (as measured by Barthel Index, or other scales), quality of life (as measured by 36-Item Short Form Health Survey), and safety.

**Conclusions:**

The results of this systematic review may summarize the up-to-date evidence on the efficacy and safety of sertraline for patients with PSD.

**Ethics and dissemination:**

This systematic review will not need any ethical approval, because it will not analyze any individual patient data. The findings of this study are expected to disseminate at peer-reviewed journals.

## 1 Introduction

Post-stroke depression (PSD) is a very frequent and serious complication for stroke survivors.^[1–3]^ Although the stroke detection is much easier for clinical practice, the identification of PSD is still challenging, because of some neurological symptoms that may conceal some primary moods.^[2]^ It has been estimated that about one-third stroke survivors experience such disorders,^[4–6]^ especially during the first stage after stroke.^[7,8]^ In fact, a recent study reported that about 85% of patients with strokes can be developed depression disorders at least 5 times within 5 years of poststroke.^[4]^ In addition, studies have reported that such condition also has close association with delayed rehabilitation, social withdrawal, and poor quality of life post the stroke.^[9–15]^

Sertraline has been reported to treat PSD effectively.^[16–24]^ However, no systematic review has performed to assess the efficacy and safety of sertraline for the treatment of PSD among the stroke survivors presently. Therefore, in this study, we will systematically evaluate the efficacy and safety of sertraline for patients with PSD.

## 2 Methods

### 2.1 Study registration

This systematic review is registered on PROSPERO (CRD42019126136). It has been reported abide to the guidelines of Preferred Reporting Items for Systematic Reviews and Meta-Analysis (PRISMA) Protocol statement.^[25]^

### 2.2 Eligibility criteria for study selection

#### 2.2.1 Types of studies

Randomized controlled trials (RCTs) of sertraline for patients with PSD will be included in this study. However, non-clinical trials, non-RCTs, quasi-RCTs, and any other studies will not be included.

#### 2.2.2 Types of interventions

The experimental treatments can be any forms of sertraline monotherapy only. The control therapies can be any interventions, except sertraline.

#### 2.2.3 Types of patients

Patients with PSD, regardless the race, sex, and age will be included in this systematic review.

### 2.3 Types of outcome measurements

#### 2.3.1 Primary outcome

Depression (as measured by Hamilton depression scale, Beck Depression Inventory or any other scales).

#### 2.3.2 Secondary outcome

Anxiety (as assessed by Hamilton anxiety scale, or other tools);

Response rate;

Activities of daily living (as measured by Barthel Index, or other scales);

Quality of life (as measured by 36-Item Short Form Health Survey);

Safety (any adverse events or reactions).

### 2.4 Search strategy

#### 2.4.1 Electronic databases sources

We will search EMBASE, MEDICINE, Web of Science, Cochrane Library, Allied and Complementary Medicine Database, Chinese Biomedical Literature Database, and China National Knowledge Infrastructure from their inceptions to the February 28, 2019. Each database will be searched without any language restrictions. We have showed the detailed search strategy for database of Cochrane Library in Table 1. Similar search strategies will be applied to all other electronic databases.

**Table 1:**
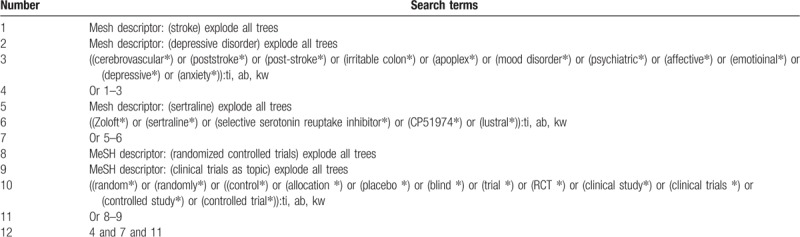
Search strategy applied in Cochrane Library database.

#### 2.4.2 Other literature sources

We will also search dissertations, ongoing trials, and conference abstracts to avoid missing any potential studies.

### 2.5 Study selection

Two reviewers will independently scan the titles and abstracts for all records in accordance with the pre-designed eligibility criteria. All studies that meet the initial eligibility criteria will be read in full texts for further selection. A third reviewer will help to resolve any disagreements arise between 2 reviewers. The process of search strategy and study selection will be presented in PRISMA study flowchart.

### 2.6 Data extraction

A standardized data extraction sheet will be utilized to collect data and important information. Two reviewers will independently carry out data extraction according to the predefined sheet. Any divergences between 2 reviewers will be solved by consulting a third reviewer. Extracted information will comprise of generation information (e.g., title, first author, published year, race, age, diagnostic criteria, etc.), Study methods (e.g., randomization, concealment, blinding, etc.), treatment details (e.g., drug, dosage, duration, etc.), and outcomes (e.g., all primary, secondary, safety outcomes).

### 2.7 Missing data dealing with

We will contact primary authors to require any insufficient or missing data from primary studies. If we can not receive those data, we will just pool the available data, and it will be discussed as a limitation.

### 2.8 Risk of bias assessment

Cochrane risk of bias tool will be used to assess the methodological quality for all eligible RCTs. This tool includes random sequence generation, allocation concealment, blinding, incomplete outcome data, selective reporting, and other biases. Each item will be further divided into 3 types, including high, unclear and low risk of bias. Two reviewers will assess the methodological quality for each eligible study independently. Any disagreements between 2 reviewers will be solved by a third reviewer through discussion.

### 2.9 Data synthesis

RevMan 5.3 (Cochrane Community, London, UK) software will be used to pool the data and to conduct meta-analysis. Mean difference or standardized mean difference and 95% confidence intervals (CIs) will be reported for continuous data, and risk ratio and 95% CIs for dichotomous data. Heterogeneity will be determined by using *I*^2^ test. When *I*^2^ ≤50%, heterogeneity is regarded acceptable, while when *I*^2^ >50%, it is considered as substantial. Meanwhile, subgroup analysis will be operated to identify possible reasons for substantial heterogeneity according to the different interventions, outcomes, and methodological quality.

When heterogeneity is acceptable, a fixed-effect model will be used to pool the data, and meta-analysis will be conducted if it is possible. When heterogeneity is significant, we will use a random-effect model to pool the data and perform meta-analysis if there is acceptable heterogeneity after subgroup analysis. Otherwise, the data will not be pooled and just narrative summary will be presented.

Additionally, sensitivity analysis will be performed for robustness check of pooled outcome results by removing low quality trials. If it is possible, funnel plot and Egger regression test will be conducted to check any possible publication bias.^[26,27]^

## 3 Discussion

PSD seriously affect quality of life in stroke survivors. Its increasing incidence also brings a heavy burden for patients, their families, and the society. Although lots of clinical studies have reported the efficacy of sertraline for patients with PSD, no study has systematically assessed its efficacy and safety. Thus, it is very necessary and important to conduct a systematic review to systematically evaluate the efficacy and safety of sertraline for PSD in stroke survivors. The results of this study will provide most recent evidence of sertraline for treating PSD for clinician and health policy makers.

## Author contributions

**Conceptualization:** Zhengfa Bai, Liu-yi Wang.

**Data curation:** Zhengfa Bai, Liu-yi Wang.

**Formal analysis:** Zhengfa Bai.

**Funding acquisition:** Liu-yi Wang.

**Investigation:** Liu-yi Wang.

**Methodology:** Zhengfa Bai, Liu-yi Wang.

**Project administration:** Liu-yi Wang.

**Resources:** Zhengfa Bai.

**Software:** Zhengfa Bai.

**Supervision:** Liu-yi Wang.

**Validation:** Zhengfa Bai, Liu-yi Wang.

**Visualization:** Zhengfa Bai, Liu-yi Wang.

**Writing – original draft:** Zhengfa Bai, Liu-yi Wang.

**Writing – review & editing:** Zhengfa Bai, Liu-yi Wang.
